# Supplementary material for: Rab22A recruits BLOC‐1 and BLOC‐2 to promote the biogenesis of recycling endosomes
Source: EMBO Rep. 2018 Nov 7;19(12):e45918. doi: 10.15252/embr.201845918 (PMC6280653; doi:10.15252/embr.201845918)
Supplement: Supplementary file 1 — Appendix [file EMBR-19-e45918-s001.pdf]

Rab22A recruits BLOC-1 and BLOC-2 to promote the biogenesis of recycling endosomes

Saurabh Shakya[1], Prerna Sharma[1*], Anshul Milap Bhatt[1*], Riddhi Atul Jani[2], Cédric Delevoye[2,3] and Subba Rao Gangi Setty[1,†]

[1]Department of Microbiology and Cell Biology, Indian Institute of Science, Bangalore, India 560012; [2]Structure and Membrane Compartments, Institut Curie, PSL Research University, CNRS, UMR 144, F-75005, Paris, France and [3]Cell and Tissue Imaging Facility (PICT-IBiSA), Institut Curie, PSL Research University, CNRS, UMR 144, F-75005, Paris, France.

'*' Both authors contributed equally to this work.
[†]Corresponding author: subba@iisc.ac.in

Table of content

Content	Page number
Appendix Table S1	2
Appendix Table S2	3

Appendix Tables

Appendix Table S1. List of TRC shRNA plasmids and their target sequences

Gene	5'-Target sequence-3'	Gene	5'-Target sequence-3'
hRab4A	sh1: GATAGAATGTGGCACTAAATC<br>sh2: CCTACAATGCGCTTACTAATC<br>sh3: TCCGTGACGAGAAGTTATTAC<br>sh4: GCTTACTTCATCAGTTTATTC	***hRab11A***	sh1: ATCATGCTGATAGTAACATTG<br>sh2: GCCTTATTGGTTTATGACATT
hRab5A	sh1: GGCAAGCAAGTCCTAACATTGC<br>sh2: CCAGGAATCAGTGTTGTAGTAC<br>sh3: GCAGCCTTCCTTTCCAAAGTTC<br>sh4: GAGAGTCCGCTGTTGGCAAATC<br>sh5: CCATAGCCTAGCACCAATGTAC	***hRab14A***	sh1: CCTCACACAATTGGTGTTGAA<br>sh2: GAAGCCAAACAGTTTGCTGAA<br>sh3: GATGCAAGGAATCTCACCAATC<br>sh4: GTGTTGAATTTGGTACAAGAA
hRab5B	sh1: GCCAGTCCTAGCATCGTTATT<br>sh2: GAAAGTCAAGCCTGGTATTAC<br>sh3: AGGTACAAGACAGCGACTTAC<br>sh4: GCAGATGACAACAGCTTATTG<br>sh5: GACCTGGCCAACAAACGTATG	***hRab22A***	sh1: GCAGCTATAATCGTTTATGAT<br>sh2: CCAAACATCAACCCAACAATA<br>sh3: CGCGATAAACATAAATGAACTC<br>sh4: CAGGTGTAGGTAAATCGAGTA<br>sh5: CAAGAACGATTTCGTGCCTTA
hRab5C	sh1: ATGCAGACGACAACAGTTTGC<br>sh2: CATTGCACTCGCGGGTAACA<br>sh3: CTTTGTCAAGGGACAGTTTC	***hMuted*** **(BLOC-1 subunit)**	sh1: GCTCAGAGAACTGTAGGTATA<br>sh2: CCACTTAGTAGCTAGTGAGAA<br>sh3: GTAGCTAGTGAGAAACAGCAT<br>sh4: GCAGCTAATGACTCAGTCTGT
hRab7A	sh1: ATGGATAAATTGCCGTTATTTC<br>sh2: ACTGCTGCGTTCTGGTATTTG<br>sh3: GGCTAGTCACAATGCAGATAT<br>sh4: ACGAATTTCCTGAACCTATCA<br>sh5: ACGTAGGCCTTCAACACAATTC<br>sh6: ACCAGTATGTGAATAAGAAAT<br>sh7: GGTTATCATCCTGGGAGATTC<br>sh8: ATAGCTGGAGAGATGAGTTTC	***hHPS6*** **(BLOC-2 subunit)**	sh1: TCACTGACACAGGAAATCATT<br>sh2: GCTTGGTCTCTCCTACAGTAA<br>sh3: GAGGGAATCTTCGTCTGCTTTC<br>sh4: GCCCATATGAGGACATCCTAT<br>sh5: GCTTCGAAGTGAAATCTTCAA
hRab9A	sh1: GACAACGGCGACTATCCTTAT<br>sh2: CCGAGGATAGGTCAGATCATT		

Appendix Table S2. List of primers used for transcript analysis

Gene	Forward and reverse primers	Amplicon size (bp)
**Human specific primers**		
hGAPDH	5'-AGTCCACTGGCGTCTTCAC-3'<br>5'-GCTGATGATCTTGAGGCTGT-3'	155
hRab4A	5'-ATCGCGGCCGCATGTCGCAGACGGCCATG-3'<br>5'-CTTCTCGAGCTAACAACCACACTCCTG-3'	657
hRab5A	5'-GGAAGATCTATGGCTAGTCGAGGCGCAA-3'<br>5'-CGCGTCGACTTAGTTACTACAACACTGA-3'	648
hRab5B	5'-GGCCATGACTAGCAGAAGTACA-3'<br>5'-AACCTCAGTTGCTACAACACTG-3'	648
hRab5C	5'-ATACATGGCGGGTCGAGGAGGT-3'<br>5'-AGCCTCAGTTGCTGCAGCACTG-3'	651
hRab7A	5'-AAGGATGACCTCTAGGAAGAAA-3'<br>5'-AGGCTCAACAACTGCAGCTTTC-3'	620
hRab9A	5'-TGGAGATGGTGGAGTTGGGA-3'<br>5'-GCAGCAGTCAGAACCTCTGT-3'	211
hRab11A	5'-GGAAGATCTATGGGCACCCGCGACGACGA-3'<br>5'-CGCGTCGACTTAGATGTTCTGACAGCACT-3'	651
hRab14A	5'-CGTGAATTCATGGCAACTGCACCATACAAC-3'<br>5'-ACGCGGATCCCTAGCAGCCACAGCCTTCTCT-3'	648
hRab22A	5'-ATGGCGCTGAGGGAGCTC-3'<br>5'-TCAGCAGCAGCTCCGCTT-3'	585
**Mouse specific primers**		
mGAPDH	5'-GAGCCAAACGGGTCATCATCT-3'<br>5'-GAGGGGCCATCCACAGTCTT-3'	220
mRab22A	5'-GCCGCTCGAGATGGCGCTGAGGGAACTT-3'<br>5'-AGGGAAGCTTTCAGCAGCAGCTTCGCTT-3'	585 bp
