# Supplementary material for: Rab22A recruits BLOC‐1 and BLOC‐2 to promote the biogenesis of recycling endosomes
Source: EMBO Rep. 2018 Nov 7;19(12):e45918. doi: 10.15252/embr.201845918 (PMC6280653; doi:10.15252/embr.201845918)
Supplement: Supplementary file 2 — Expanded View Figures PDF [file EMBR-19-e45918-s002.pdf]

Expanded View Figures

Figure EV1. Analysis of endosomal Rab RNAi screen and the localization of Rabs to REs.

A IFM images of HeLa cells to study the localization of KIF13A-YFP to REs.
B PCR analysis of Rab-knockdown HeLa cells to measure the knockdown efficiency of each shRNA as indicated. DNA band intensities were quantified and indicated on the gels.
C Subcellular fractionation of HeLa cells to study the localization of different Rabs (red box) with respect to STX13 or AP-1.
D, E IFM analysis of HeLa cells that were transfected with different constructs as indicated.
F Live cell imaging of mCherry-Rab22A with respect to GFP-Rab9A in HeLa cells. Arrowheads point to the Rab22A-positive tubular structures. Magnified view of insets (at 0, 16, 40 s) are shown separately. Scale bars: 10 μm.
G IFM images of Rab22A sh, BLOC-1 sh and control sh HeLa cells that were transfected with indicated constructs.
H Graphs represent the measurement of corrected total cell fluorescence (CTCF) in HeLa cells of Fig 2A. n = 6–8 cells. Average CTCF values (AU: arbitrary units) and their respective fold changes (mean ± SEM) are indicated.

Data information: In (A), arrowheads and arrows point to the KIF13A-positive tubular REs and E/SEs, respectively. In (D, E, G), arrowheads point to the STX13- or KIF13A-positive tubular REs or E/SEs. Scale bars: 10 μm.

 © 2018 The Authors

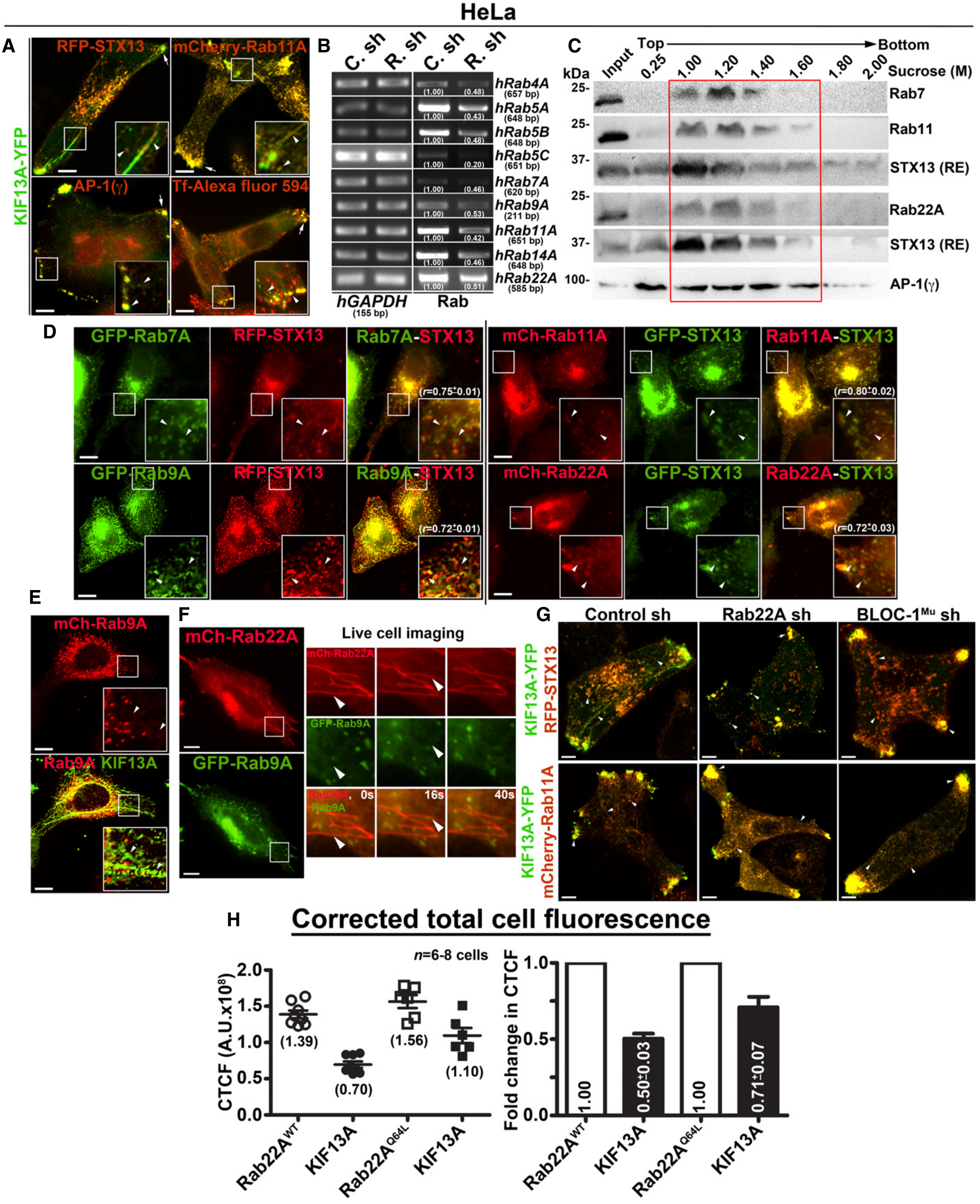
HeLa
A
RFP-STX13
mCherry-Rab11A
AP-1(γ)
Tf-Alexa fluor 594
KIF13A-YFP
B
C. sh
R. sh
hRab4A (657 bp)
hRab5A (648 bp)
hRab5B (648 bp)
hRab5C (651 bp)
hRab7A (620 bp)
hRab9A (211 bp)
hRab11A (651 bp)
hRab14A (648 bp)
hRab22A (585 bp)
hGAPDH (155 bp)
Rab
C
Top
Bottom
kDa
Input
0.25
1.00
1.20
1.40
1.60
1.80
2.00
Sucrose (M)
Rab7
Rab11
STX13 (RE)
Rab22A
AP-1(γ)
D
GFP-Rab7A
RFP-STX13
Rab7A-STX13
(r=0.75±0.01)
mCh-Rab11A
GFP-STX13
Rab11A-STX13
(r=0.80±0.02)
GFP-Rab9A
Rab9A-STX13
(r=0.72±0.01)
mCh-Rab22A
Rab22A-STX13
(r=0.72±0.03)
E
mCh-Rab9A
Rab9A KIF13A
F
mCh-Rab22A
GFP-Rab9A
Live cell imaging
0s
16s
40s
G
Control sh
Rab22A sh
BLOC-1Mu sh
KIF13A-YFP RFP-STX13
KIF13A-YFP mCherry-Rab11A
H
Corrected total cell fluorescence
n=6-8 cells
CTCF (A.U.x10⁸)
(1.39)
(0.70)
(1.56)
(1.10)
Fold change in CTCF
1.00
0.50±0.03
1.00
0.71±0.07
Rab22AWT
KIF13A
Rab22AQ64L
KIF13A

Figure EV1.

Figure EV2. Analysis of mouse melanocytes expressing different constructs of Rab22A.

A Live cell imaging of mCherry-Rab22A with respect to GFP-STX13 in wild-type melanocytes. Arrows point to Rab22A-positive buds or tubular structures arising from STX13-positive E/SEs (arrowheads). Magnified view of insets (at various seconds) is shown separately. Scale bars: 10 μm.

B IFM analysis of mCherry-Rab22A$^{WT/Q64L/S19N}$-transfected melanocytes. Arrowheads point to Rab22A localization, and arrows indicate the melanocyte pigmentation. Nuclei are stained with Hoechst 33258. The colocalization coefficient (r, in mean ± SEM) between two markers was indicated separately. Scale bars: 10 μm.

C, D Quantification of melanin content (C, n = 5) and the protein levels of TYRP1 (judged by immunoblotting in D, n = 3) in melanocytes that were transfected with GFP-Rab22A$^{WT/Q64L/S19N}$. In (D), graph indicates the relative levels (mean ± SEM) of TYRP1 with respect to overexpressed Rab22A. Protein band intensities were quantified and indicated on the gels. **$P \leq 0.01$, ***$P \leq 0.001$ and ns = not significant (unpaired Student's t-test).

 © 2018 The Authors

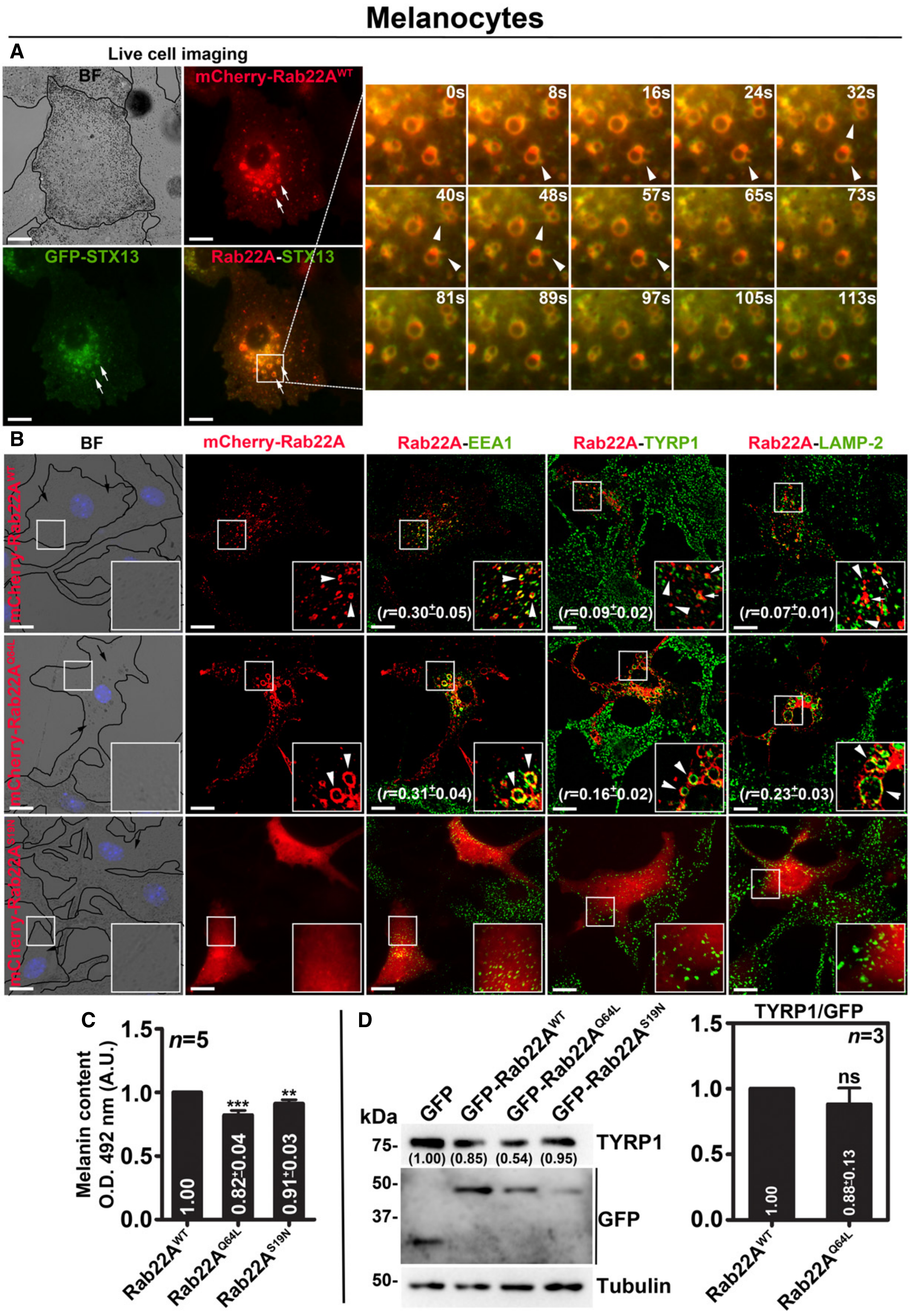
Melanocytes
A
Live cell imaging
BF
mCherry-Rab22A^WT
GFP-STX13
Rab22A-STX13
0s
8s
16s
24s
32s
40s
48s
57s
65s
73s
81s
89s
97s
105s
113s
B
BF
mCherry-Rab22A
Rab22A-EEA1
Rab22A-TYRP1
Rab22A-LAMP-2
mCherry-Rab22A^WT
(r=0.30±0.05)
(r=0.09±0.02)
(r=0.07±0.01)
mCherry-Rab22A^Q64L
(r=0.31±0.04)
(r=0.16±0.02)
(r=0.23±0.03)
mCherry-Rab22A^S19N
C
n=5
Melanin content O.D. 492 nm (A.U.)
1.00
0.82±0.04
0.91±0.03
***
**
Rab22A^WT
Rab22A^Q64L
Rab22A^S19N
D
kDa
GFP
GFP-Rab22A^WT
GFP-Rab22A^Q64L
GFP-Rab22A^S19N
75-
(1.00) (0.85) (0.54) (0.95)
TYRP1
50-
37-
GFP
50-
Tubulin
TYRP1/GFP
n=3
ns
1.00
0.88±0.13
Rab22A^WT
Rab22A^Q64L

Figure EV2.

Figure EV3. Analysis of cargo recycling in control and Rab22A-, BLOC-1-, BLOC-2-knockdown HeLa cells.

A IFM images of KIF13A-YFP-transfected control and knockdown HeLa cells as indicated. Arrowheads point to the localization of KIF13A to E/SEs or REs.
B Membrane-cytosol fractionation of HeLa cell homogenate for the localization of KIF13A. *, non-specific bands.
C IFM images of control and Rab22A-, BLOC-1-knockdown HeLa cells that were stained with LAMP-2 or internalized with fluorescein–dextran.
D Cell surface levels of LAMP-1 and M6PR in control and Rab22A-, BLOC-1-knockdown HeLa cells measured using flow cytometry. Normalized mean fluorescence intensity (MFI) was calculated (mean ± SEM) and then plotted. $n = 3$.
E IFM images of HeLa cells that were subjected to Tf-Alexa Fluor 594 recycling kinetics.
F Fluorescence intensities Tf in the images of (E) were quantified and plotted (mean ± SEM). $n = 3$. n_c = total number of cells.
G Immunoblotting analysis of Tf receptor in HeLa cells as indicated. Protein band intensities were quantified and indicated on the gels.

Data information: In (A, C, E), arrows point to the localization of cytoskeletal proteins (A) or internalized dextran or lysosomes (C) or accumulation of Tf to the intracellular vesicles (E). Scale bars: 10 μm. In (D, F), $*P \leq 0.05$, $**P \leq 0.01$, $***P \leq 0.001$ and ns = not significant (unpaired Student's *t*-test).

 © 2018 The Authors

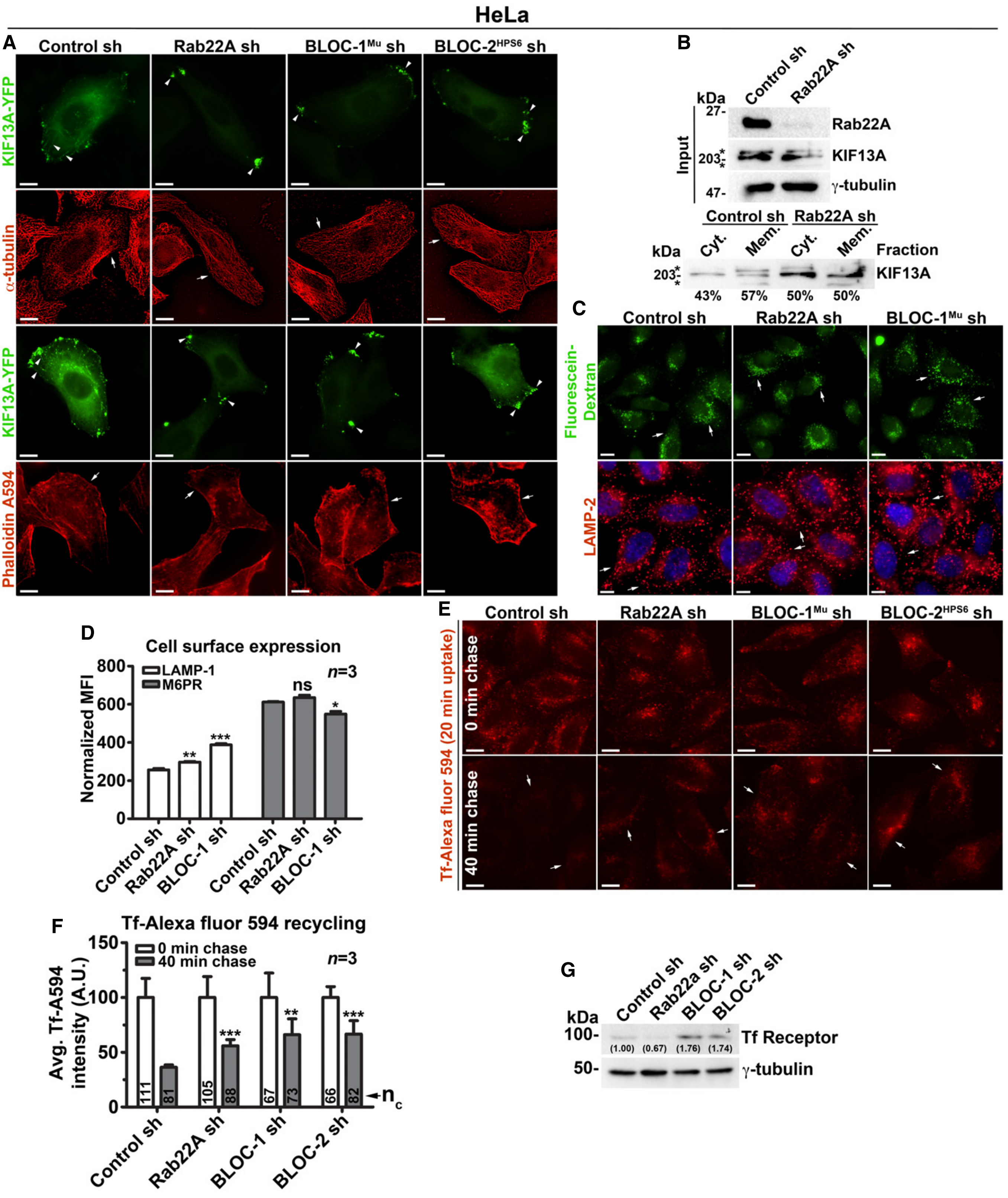
HeLa
A
Control sh
Rab22A sh
BLOC-1Mu sh
BLOC-2HPS6 sh
KIF13A-YFP
α-tubulin
KIF13A-YFP
Phalloidin A594
B
kDa
Control sh
Rab22A sh
27-
Rab22A
Input
203
KIF13A
47-
γ-tubulin
Control sh
Rab22A sh
kDa
Cyt.
Mem.
Cyt.
Mem.
Fraction
203
KIF13A
43%
57%
50%
50%
C
Control sh
Rab22A sh
BLOC-1Mu sh
Fluorescein-Dextran
LAMP-2
D
Cell surface expression
LAMP-1
M6PR
n=3
Normalized MFI
800
600
400
200
0
ns
Control sh
Rab22A sh
BLOC-1 sh
Control sh
Rab22A sh
BLOC-1 sh
E
Control sh
Rab22A sh
BLOC-1Mu sh
BLOC-2HPS6 sh
Tf-Alexa fluor 594 (20 min uptake)
0 min chase
40 min chase
F
Tf-Alexa fluor 594 recycling
0 min chase
40 min chase
n=3
Avg. Tf-A594 intensity (A.U.)
150
100
50
0
111
81
105
88
67
73
66
82
nc
Control sh
Rab22A sh
BLOC-1 sh
BLOC-2 sh
G
kDa
Control sh
Rab22a sh
BLOC-1 sh
BLOC-2 sh
100-
(1.00)
(0.67)
(1.76)
(1.74)
Tf Receptor
50-
γ-tubulin

Figure EV3.

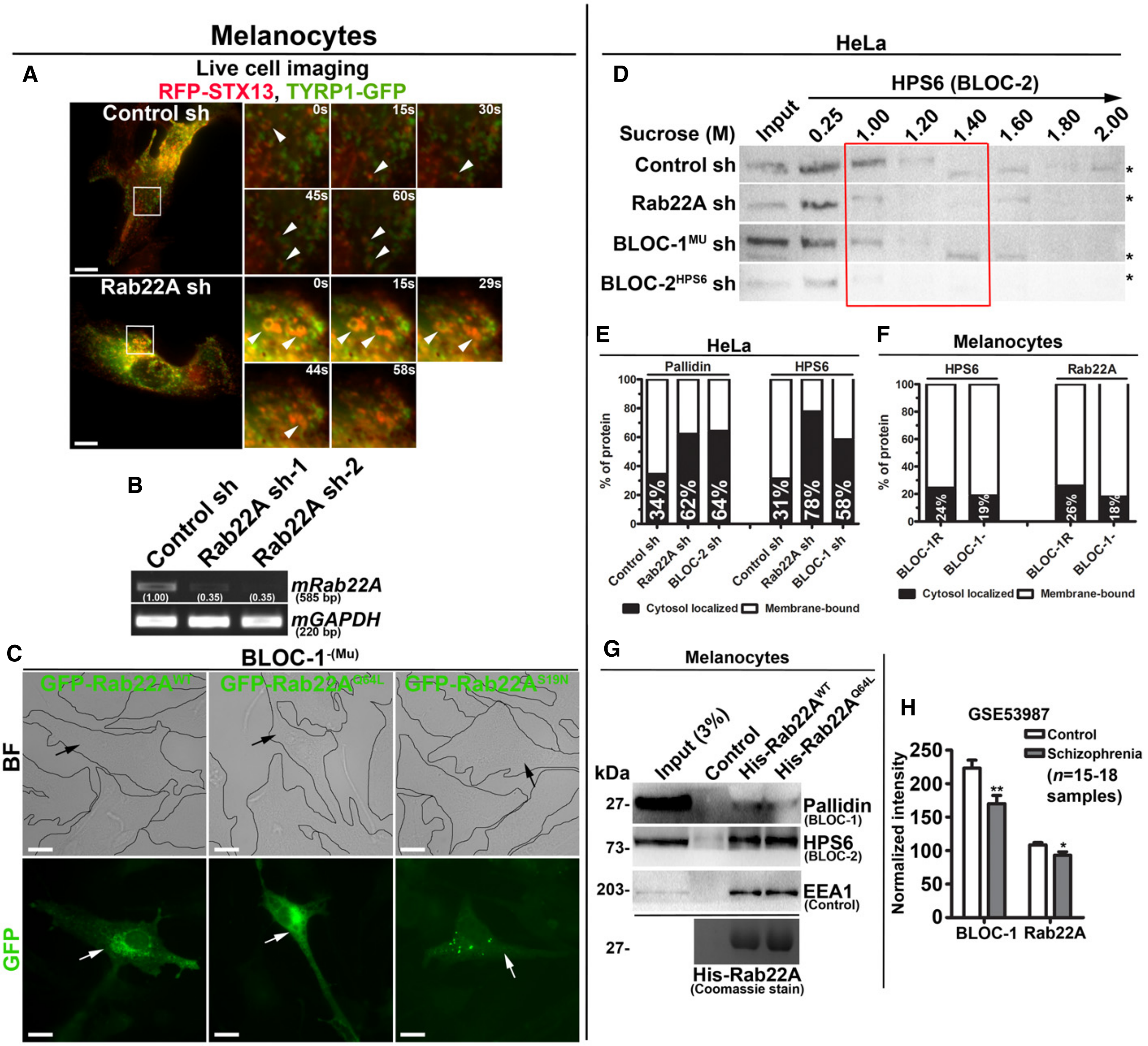

Figure EV4. Analysis of Rab22A-knockdown and BLOC-1-deficient melanocytes expressing different constructs and membrane association and interaction studies of Rab22A. Rab22A as possible susceptible gene for schizophrenia.

- A Live cell imaging of TYRP1-GFP with respect to RFP-STX13 in control and Rab22A-depleted melanocytes. Arrowheads point to TYRP1-positive structures. Magnified view of insets (at various seconds) is shown separately. Scale bars: 10 µm.
- B PCR analysis of Rab22A-depleted melanocytes. DNA band intensities were quantified and indicated on the gels.
- C BF and IFM images of GFP-Rab22A$^{WT/Q64L/S19N}$-transfected BLOC-1-deficient melanocytes. Black and white arrows indicate the melanocyte pigmentation and GFP-Rab22A expression, respectively. Scale bars: 10 µm.
- D Subcellular membrane fractionation of control and knockdown HeLa cells. The fractions were probed for HPS6 (red box).
- E, F Graphs represent the percentage of membrane association of pallidin or HPS6 or Rab22A in the indicated cell types and derived from Fig 5B and C, respectively. $n = 1$.
- G Pull-down of His-Rab22A$^{WT/Q64L}$ using melanocyte lysate. The bead-bound His-Rab22A was shown as coomassie-stained gel separately.
- H Transcript levels of Rab22A and pallidin (BLOC-1) in control and schizophrenia patients. Analysed from data set GSE53987 and then plotted (mean ± SEM). n = 15–18 samples. *$P \leq 0.05$ and **$P \leq 0.01$ (unpaired Student's t-test).

© 2018 The Authors
