# Supplementary material for: Rab22A recruits BLOC‐1 and BLOC‐2 to promote the biogenesis of recycling endosomes
Source: EMBO Rep. 2018 Nov 7;19(12):e45918. doi: 10.15252/embr.201845918 (PMC6280653; doi:10.15252/embr.201845918)
Supplement: Supplementary file 3 — Movie EV1 [file EMBR-19-e45918-s003.zip › MovieEV1/MovieEV1_legend.docx]

**Movie EV1.** Time-lapse imaging of GFP-Rab22A and mCherry-Rab11A in HeLa cells.
