# Supplementary material for: Rab22A recruits BLOC‐1 and BLOC‐2 to promote the biogenesis of recycling endosomes
Source: EMBO Rep. 2018 Nov 7;19(12):e45918. doi: 10.15252/embr.201845918 (PMC6280653; doi:10.15252/embr.201845918)
Supplement: Supplementary file 6 — Movie EV4 [file EMBR-19-e45918-s006.zip › MovieEV4/MovieEV4_legend.docx]

**Movie EV4.** Time-lapse imaging of mCherry-Rab22A^WT^ and GFP-STX13 in wild-type (melan-Ink4a) melanocytes.
